# Policy Intervention and Financial Sustainability in an Emerging Economy: A Structural Vector Auto Regression Analysis

**DOI:** 10.3389/fpsyg.2022.924545

**Published:** 2022-08-05

**Authors:** Sarah Ahmed, Nazima Ellahi, Ajmal Waheed, Nida Aman

**Affiliations:** ^1^Department of Business Administration, Foundation University Islamabad, Islamabad, Pakistan; ^2^Department of Economics and Finance, Foundation University Islamabad, Islamabad, Pakistan; ^3^Faculty of Management Sciences, Foundation University Islamabad, Islamabad, Pakistan; ^4^Management Sciences, Bahria University, Islamabad, Pakistan

**Keywords:** SVAR, R software, financial sustainability, impulse response function, financialization, firm performance, corporate governance, sectoral policies

## Abstract

The purpose of the study is to observe the impact of policy intervention on financial sustainability using the structural vector autoregression (SVAR) analysis. The population of the study is the manufacturing sector of Pakistan, which is an emerging economy. Data for 249 firms operating in the manufacturing sector are taken, collected from Datastream from 2005 to 2019, with total observations of 2,400. To conduct the analysis, R software is used for its better visualization. Results show that firm performance, corporate governance, and sectoral policies have a positive and long-term impact on financial sustainability, whereas earning management and financialization not only have a negative impact, but this impact affects the operations of the corporate for a longer period. This study would be helpful for policymakers as it gives a framework for financial sustainability based on the policies and strategies developed by the sector.

## Introduction

Financial crises over the years have altered the way corporates were operating and used to make their policies (Aqeel et al., 2021a,b, 2022; Li et al., 2021; Wang et al., 2021; Zhou et al., 2021; NeJhaddadgar et al., 2022). Now, corporates enter the market to stay without becoming a going concern (Abbas et al., 2021; Mubeen et al., 2021a,b; Aman et al., 2022; Ge et al., 2022; Liu et al., 2022). They make such strategies that help them function effectively by taking care of all the stakeholders (Abbas et al., 2019b; Hussain et al., 2019, 2021; Mamirkulova et al., 2020; Mubeen et al., 2020, 2021a,b; Abbasi et al., 2021; Azizi et al., 2021; Mamirkulova and Mi, 2022). These financial crises have also taught them that working to get short-term goals and increasing the worth of shareholders can damage in the longer run (Anjum et al., 2017; Abbas, 2020; Maqsood et al., 2021; Raza Abbasi et al., 2021; Rahmat et al., 2022). These shifting trends have instigated an inescapable responsibility for corporates to look after all the stakeholders if they want to be successful and become financially sustainable (Zhang et al., 2012; Asad et al., 2017; Abbas et al., 2019b; Aman et al., 2019; Irfan and Ali, 2019).

Corporates operating in developed nations are far ahead in adopting policies that can help them in sustainable development as compared to the ones working in developing nations (Ali and Irfan, 2020). Input from developing nations is limited in sustainability (Alshehhi et al., 2018) and (Shah et al., 2016), and research related to financial sustainability is also limited (Zabolotnyy and Wasilewski, 2019). This article is an attempt to see the policy interventions of corporates operating in the manufacturing sector of Pakistan to gain financial sustainability by using structural vector autoregression (SVAR). In addition, structural impact on corporate policies devised and implemented from time to time is also carried out by applying SVAR.

## Review of Literature

Structural vector autoregression^1^ models allow the contemporaneous interdependencies between the left-hand-side variables (Deleidi et al., 2021). SVAR models can identify shocks with the help of the Impulse Response Analysis (IRA) or restrictions can be applied by the theory (Yang et al., 2022). It is useful to analyze the dynamics of the model by introducing it to unexpected shocks. The default SVAR model was a scoring algorithm by Amisano and Giannini (1997). The SVAR models are implemented through various software packages like EViews, R, etc. which makes the use of SVAR models simple.

In SVAR models, impulse response functions (IRFs) are usually computed to see the response of the model to a deviation or a shock (Gottschalk, 2001). SVAR models are structural models, which are used to explore the dynamic behavior of the variables from the theoretical viewpoint or a theory-guided view. VAR and SVAR are used in research to address problems of endogeneity and causality. In one study, VAR was used to examine the impact of the growth of public debt in Nigeria. It was observed that debt has a positive impact on the economic growth rate a bidirectional link exists between public debt and economic growth (Egbetunde, 2012). In another study panel, VAR was used to analyze the causal relationship between government debt and economic growth for a sample of 20 developed countries and found a negative relation between the two variables (Lof and Malinen, 2014). Bal et al. (2022) used SVAR to analyze the impact of electricity consumption on economic growth.

Sustainability has become a new mantra for the current century. This concept originated from the 1987 Brundtland Report. The concept encompasses three dimensions: social, economic, and environmental. The pursuit of economic and social impartiality has been a real aim for the past many years. The importance of economic well-being is a renowned phenomenon, but the issue with this economic or financial well-being is to take the output toward a longer time frame where future generations can also benefit from it.

## Methodology

For this purpose, structural VAR is used, with the help of IRF, the future intervention of the variables is studied and shocks from each exogenous variable are identified as well. This study followed research procedures for analysis (Yoosefi Lebni et al., 2020; Aman et al., 2021; Khazaie et al., 2021; Moradi et al., 2021; Paulson et al., 2021; Farzadfar et al., 2022). This analysis aids to pinpoint the direction and strength of the impact from each variable. SVAR is conducted to observe the future intervention of the variables, which are part of the framework. The analysis is performed in R software that cannot only help to analyze the data but would also assist in better visualization of the policy interventions (Khattak et al., 2013; Malik et al., 2017; Asad et al., 2020a,b; Ashraf et al., 2020; Zhu et al., 2021). R software is used because of the following reasons, these reasons are also justified by Incerti et al. (2019) and Mizumoto and Plonsky (2016). R software is used because of its cost-effectiveness. It is open-source software and all the packages in R software are also easily available on the internet. Moreover, it is updated by the researchers frequently and the updated version is shared openly. That helps to access the latest syntax.

The SVAR model is used to identify the shocks and trace them out (Pfaff, 2008). Moreover, it is used to identify short-term or long-term policy interventions. Impulse response function IRF helps to identify the shocks caused by each variable and the intensity of the shock as well. The impact in the form of shocks can be seen over the sample period. The packages used for PVAR, and SVAR are vars, urca, plm, panel var, and dev tools.

For the study, firm performance, financialization, corporate governance, policies, and earning management are taken as the independent variables and financial sustainability is the dependent variable as shown in Table 1.

**TABLE 1:** Variables and their determinants.

VARIABLE	Measures	Formula
Financial sustainability (FS)	Revenue ratio	Cost of revenue/_*Total*_ revenue
	Net financial liabilities	Log (short-term debt + long-term debt – cash and cash equivalents)
	Total debt services cover	EBIT/total debt
	Cash expense cover	Total cash/interest expense
	Asset sustainability	Capital expenditure on replacement of assets/depreciation expenditure
	SGR	Retention rate * ROE
Firm performance (FP)	Tobin’s Q	(Market value of equity + book value of debt)/book value of assets
	Operating ratio	Operating profit/sales
	Net debt activity ratio	Debt/total assets
Financialization	Financialization	Long term investment + short term investment)/ total assets
Corporate governance	CG	Index of (CEO duality, institutional ownership, board meeting, board size, director ownership, audit committee, audit committee size, big 5 ownership, auditing by big 4)
Sectoral policies	SP	Dummy variable 0 and 1. 0 if policy is not applied and 1 otherwise
Control variables	Firm age	Number of years firm is incorporated
	Taxation	
Earnings management	Accrual earnings management (AEM) Real earnings management (REM)	Dechow method Cash flow from operations/total assets of previous year = 1/total assets previous year + sales revenue/total assets previous years + change in sales/total assets previous year

The econometric model of the study is:


$$\begin{array}{l} FS_{it}={\text{β}}_{0}+{\text{β}}_{1}\;FiP_{it}+{\text{β}}_{2}FIN_{it}+{\text{β}}_{3}EM_{it}+{\text{β}}_{4}SP_{it}+{\text{β}}_{5}CGI_{it}\\ +\;{\text{β}}_{6}\;Firm\;Age_{it}+{\text{β}}_{7}Tax_{it}+{\text{ε}}_{it} \end{array}$$

Where FS is the financial sustainability, FiP is the firm performance, EM is the earning management, SP is the sectoral policies, CGI is the corporate governance index, and firm age and tax are taken as the control variables.

Six models are tested with the help of SVAR. These six models are based on the six determinants of FS financial sustainability. The determinants are explained in the table above. The models for testing are:

**Model 1:**


$$\begin{array}{l} \text{Revenue ratio}={\text{β}}_{0}+{\text{β}}_{1}Tobin'sQ_{it}+{\text{β}}_{2}FIN_{it}+{\text{β}}_{3}AEM_{it}\\ +{\text{β}}_{4}SP_{it}+{\text{β}}_{5}CGI_{it}+{\text{β}}_{6}\;Firm\;Size_{it}+{\text{β}}_{8}\;Firm\;Age_{it}\\ +{\text{β}}_{9}Tax_{it}+{\text{β}}_{10}REM_{it}+{\text{β}}_{11}\;Operating\;ratio_{it}+{\text{β}}_{12}\;Net\;debt\\ activity\;ratio_{it}+{\text{β}}_{13}\;Net\;equity\;ratio_{it}+{\text{ε}}_{it} \end{array}$$

**Model 2:**


$$\begin{array}{l} \text{Net financial liabilities}=\beta_{0}+\beta_{1}Tobin'sQ_{it}+\beta_{2}FIN_{it}\\ +\beta_{3}AEM_{it}+\beta_{4}SP_{it}+\beta_{5}CGI_{it}+\beta_{6}\;Firm\;Size_{it}+\beta_{8}\;Firm\;Age_{it}\\ +\beta_{9}Tax_{it}+\beta_{10}REM_{it}+\beta_{11}\;Operating\;ratio_{it}+\beta_{12}\;Net\;debt\\ \;activity\;ratio_{it}+\beta_{13}\;Net\;equity\;ratio_{it}+\varepsilon_{it} \end{array}$$

**Model 3:**


$$\begin{array}{l} \text{Net debt service cover}={\text{β}}_{0}+{\text{β}}_{1}Tobin'sQ_{it}+{\text{β}}_{2}FIN_{it}\\ +{\text{β}}_{3}AEM_{it}+{\text{β}}_{4}SP_{it}+{\text{β}}_{5}CGI_{it}+{\text{β}}_{6}\;Firm\;Size_{it}+{\text{β}}_{8}\;Firm\;Age_{it}\\ +{\text{β}}_{9}Tax_{it}+{\text{β}}_{10}REM_{it}+{\text{β}}_{11}\;Operating\;ratio_{it}+{\text{β}}_{12}\;Net\;debt\\ activity\;ratio_{it}+{\text{β}}_{13}\;Net\;equity\;ratio_{it}+{\text{ε}}_{it} \end{array}$$

**Model 4:**


$$\begin{array}{l} \text{Cash expense cover ratio}={\text{β}}_{0}+{\text{β}}_{1}Tobin'sQ_{it}+{\text{β}}_{2}FIN_{it}\\ +{\text{β}}_{3}AEM_{it}+{\text{β}}_{4}SP_{it}+{\text{β}}_{5}CGI_{it}+{\text{β}}_{6}\;Firm\;Size_{it}+{\text{β}}_{8}\;Firm\;Age_{it}\\ +{\text{β}}_{9}Tax_{it+}{\text{β}}_{10}REM_{it}+{\text{β}}_{11}\;Operating\;ratio_{it}+{\text{β}}_{12}\;Net\;debt\\ activity\;ratio_{it}+{\text{β}}_{13}\;Net\;equity\;ratio_{it}+{\text{ε}}_{it} \end{array}$$

**Model 5:**


$$\begin{array}{l} \text{Asset sustainability}={\text{β}}_{0}+{\text{β}}_{1}Tobin'sQ_{it}+{\text{β}}_{2}FIN_{it}\\ +{\text{β}}_{3}AEM_{it}+{\text{β}}_{4}SP_{it}+{\text{β}}_{5}CGI_{it}+{\text{β}}_{6}\;Firm\;Size_{it}+{\text{β}}_{8}\;Firm\;Age_{it}\\ +{\text{β}}_{9}Tax_{it}+{\text{β}}_{10}REM_{it}+{\text{β}}_{11}\;Operating\;ratio_{it}+{\text{β}}_{12}\;Net\;debt\\ activity\;ratio_{it}+{\text{β}}_{13}\;Net\;equity\;ratio_{it}+{\text{ε}}_{it} \end{array}$$

**Model 6:**


$$\begin{array}{l} \text{SGR}={\text{β}}_{0}+{\text{β}}_{1}Tobin'sQ_{it}+{\text{β}}_{2}FIN_{it}+{\text{β}}_{3}AEM_{it}+{\text{β}}_{4}SP_{it}\\ +{\text{β}}_{5}CGI_{it}+{\text{β}}_{6}\;Firm\;Size_{it}+{\text{β}}_{7}\;Firm\;Age_{it}+{\text{β}}_{9}Tax_{it}+{\text{β}}_{10}REM_{it}\\ +{\text{β}}_{11}\;Operating\;ratio_{it}+{\text{β}}_{12}\;Net\;debt\;activity\;ratio_{it}\\ +{\text{β}}_{13}\;Net\;equity\;ratio_{it}+{\text{ε}}_{it} \end{array}$$

Table 2 shows the number of firms operating in the manufacturing sector and the number of firms from which data are collected.

**TABLE 2:** Actual number of firms and number of firms whose data is available.

Sub-sectors	Actual number	Data collected	Sub-sectors	Actual number	Data collected
Auto	21	18	Refinery	4	3
Cement	22	18	Sugar and allied	29	18
Chemical	28	22	Synthetic and rayon	10	7
Cable and electrical goods	6	4	Technology and communication	12	5
Engineering	9	9	Miscellaneous	11	11
Fertilizer	6	6	Textile composite	56	21
Food and personal care	22	14	Textile spinning	69	35
Glass and ceramics	10	7	Textile weaving	11	6
Leather	5	2	Tobacco	3	2
Oil and gas	13	08	Transport	5	4
Paper and board	10	6	Vanaspati and allied	6	2
Pharmaceuticals	12	09	Woolen	2	1
Power generation and distribution	17	11	Total	379	249

Data is collected from 2005 to 2019 for 249 firms and excluding the missing and the unavailable data the total count of observations is over 2400.

## Analysis

To see the impact of each variable on the endogenous variable, structural vector auto-regression SVAR is carried out. Structural vector autoregression helps to identify the structural shocks and how those shocks would behave over some time. The intensity and the duration of impact can be visually represented also, as done in the following section.

The R software is used for the analysis, and the package SVARs are installed for the purpose. Structural VAR applies to each model. The variable of interest in the study is financial sustainability, which is studied through different models. The basic purpose of the study is to identify the elements, which help the firm attain financial stability in the longer run. Based on the purpose of the study the restriction imposed on SVAR is BQ or Blanchard and Quah restriction, proposed by Blanchard and Quah (1988), which states imposing restrictions on how stocks influence the endogenous variables in the long run and limiting the response from a variable to a shock. The syntax used to calculate the SVAR in R is:

**SVAR estimation:**

Model6 ← VAR (dset6, *p* = 4, type = “const”)

SVAR6 ← BQ (Model6)

summary (SVAR6).

**Impulse response function:**

irf6 ← irf (SVAR6, impulse = “financialization1,”

response = “log.revratio,” n.ahead = 10)

plot(irf6).

The IRF generated is shown here for all the variables and all the models to get a clear idea of the shocks and behavior of the variables over the period. Estimates for all the models are given as under, showing the values for 10 years as the data is yearly in terms of the estimated contemporaneous impact matrix, estimated identified long-run impact matrix, and covariance matrix of reduced form residuals. The visualization of the SVAR model is also shown:

### Model 1

**FIGURE 1:**
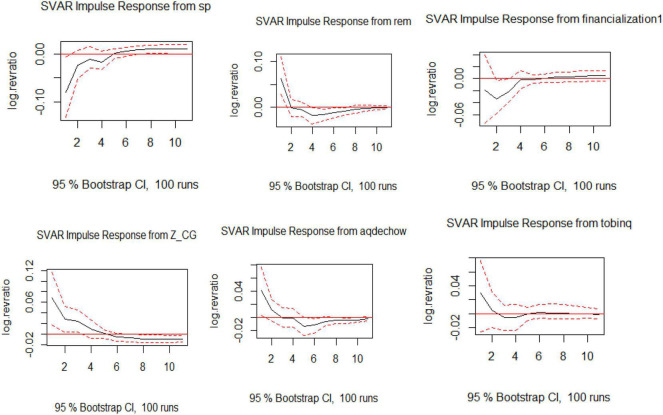
Visualization of impulse response function (IRF) for structural vector autoregression (SVAR) for Model 1.

Based on the visualization as presented in Figure 1, the graphs show the effect of each exogenous variable on the endogenous variable. Sectoral policies are showing an upward trend in future years to come, which shows the efficacy of the policies instigated in the various sectors under study. Moreover, the negative impact of financialization subsides over a couple of years and, afterward, stabilizes in the later years. The effect of REM and AEM is very damaging for the revenue ratio of firms, as the impact is a deep plunge that stays the same in the longer term.

### Model 2

**FIGURE 2:**
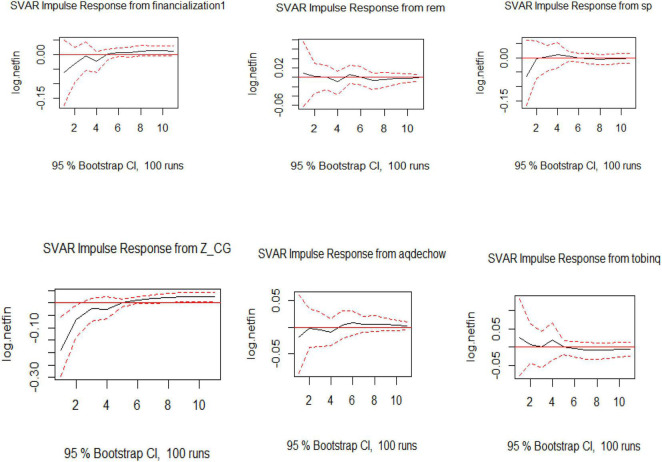
Visualization of IRF for SVAR for Model 2.

The SVAR analysis is conducted on Model 2 to see the long-term shocks of the variables on the endogenous variable, which is the net financial liability. The dependent variables are financialization, real earning management, accrual earning management, Tobin Q, and sectoral policies. Over 10 years, net financial liability is affected by exogenous variables, but the trend settles down in the longer term. Sectoral policies SP, take the net financial liability upward initially, and then, there is a stabilized effect on the endogenous variable. This shows that the policies developed by sectors are effective and working in a positive direction. Similarly, the impact shown by Tobin Q is downward initially till year 3, then there is a rise in year 4, after which the impact has stabilized in the later years as shown in Figure 2.

### Model 3

**FIGURE 3:**
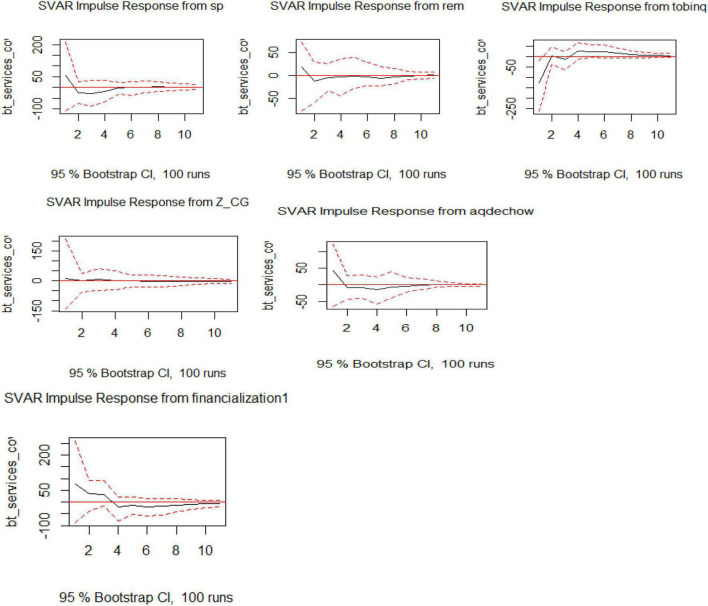
Visualization of IRF for SVAR for Model 3.

Visualization of SVAR for Model 3 shows the impact of exogenous variables on an endogenous variable, which is debt services cover. The impact of Tobin Q is taking the endogenous variable upward and this hike then persists in the years to come. The impact of REM, AEM, financialization, and SP are downward, and the decline becomes part of the trend in the later years as shown in Figure 3.

### Model 4

**FIGURE 4:**
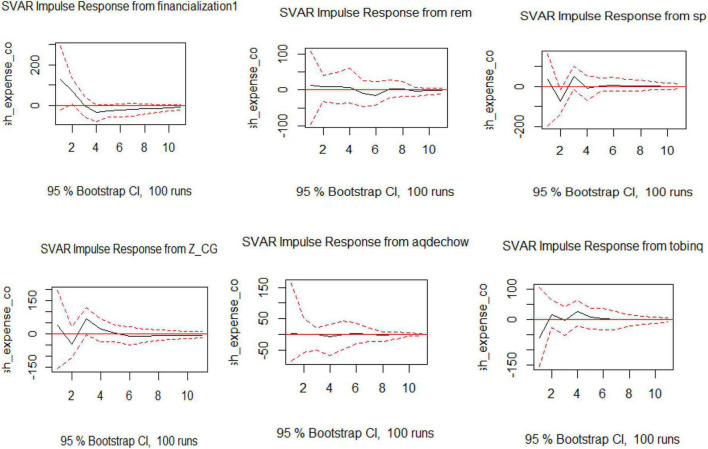
Visualization of IRF for SVAR for Model 4.

The IRF shows the impact of the exogenous variable on cash expense cover as part of Model 4. The IRF of SVAR shows financialization has a great negative impact, which is long-lasting, and firms cannot return to their initial position because of financialization. REM and AEM are not affected gravely; whereas the Tobin Q is showing a positive impact and the influence is for a longer period. Furthermore, SP is keeping a stabilized pattern over the time frame as shown in Figure 4.

### Model 5

**FIGURE 5:**
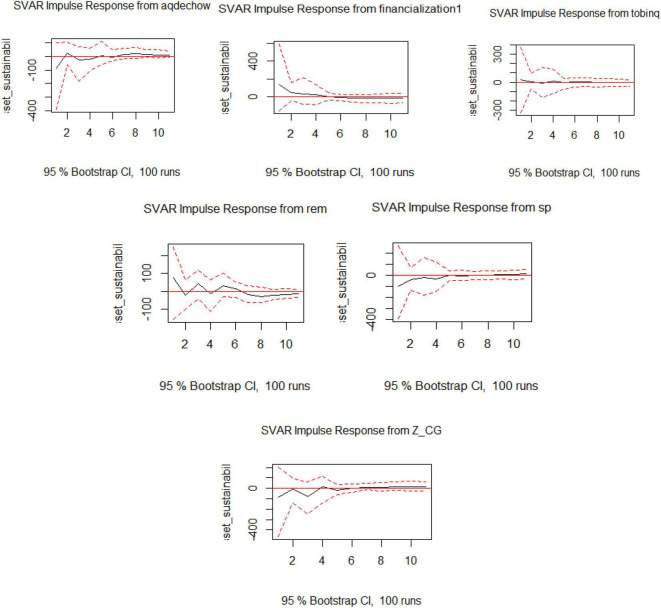
Visualization of IRF for SVAR for Model 5.

The IRF shows the impact of the exogenous variable on asset sustainability as part of Model 5. The impact of financialization is downward sloping and its impact continues for the longer term. The influence of REM and AEM is not very damaging, as the upward trend is balanced out by a downward trend of the same effect. The effect of SP is also stabilized over the period as shown in Figure 5.

### Model 6

**FIGURE 6:**
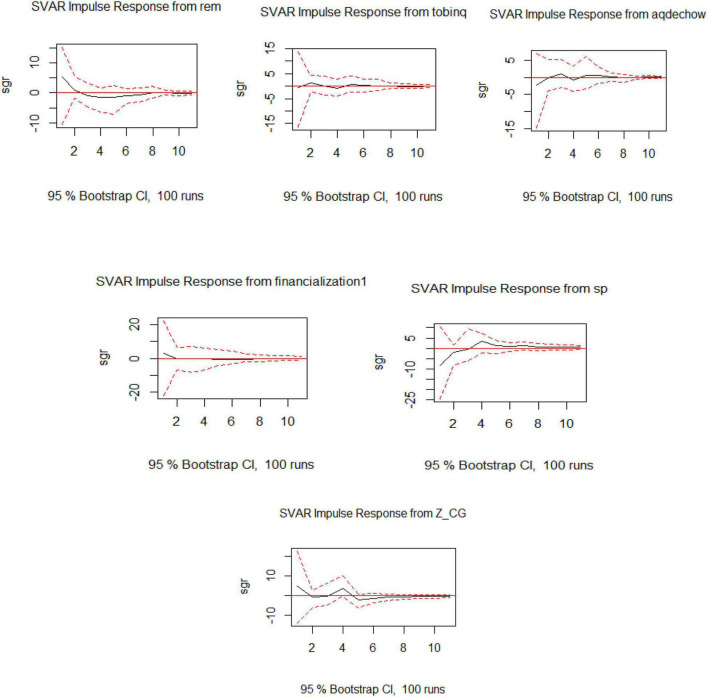
Visualization of IRF for SVAR for Model 6.

The IRF shows the impact of the exogenous variable on SGR as part of Model 6. The impact on SGR from the exogenous variables is not very intense and stabilizes in the longer run. The downward trend from REM and AEM even outs in a year or two to balance the impression. The effect of SP is upward, which shows the effectiveness of the sectoral policies as shown in Figure 6.

The SVAR analysis shows that firm performance, corporate governance, and sectoral policies have a positive and long-lasting positive impact on financial sustainability (Badar and Irfan, 2018; Asad et al., 2020a,b; Yan et al., 2020; Ali et al., 2021; Zhu et al., 2021), whereas earning management and financialization have a negative impact that affects financial sustainability for the years to come.

## Conclusion

The basic aim of a corporation is to stay in the market for a longer period. For that, they make strategies and policies for sustainable development, but not all the policies, as clear from the past, are fruitful for industrial growth. This study conducts a policy intervention anals.

## Data Availability Statement

The original contributions presented in this study are included in the article/supplementary material, further inquiries can be directed to the corresponding author.

## Author Contributions

All authors listed have made a substantial, direct, and intellectual contribution to the work, and approved it for publication.

## Conflict of Interest

The authors declare that the research was conducted in the absence of any commercial or financial relationships that could be construed as a potential conflict of interest.

## Publisher’s Note

All claims expressed in this article are solely those of the authors and do not necessarily represent those of their affiliated organizations, or those of the publisher, the editors and the reviewers. Any product that may be evaluated in this article, or claim that may be made by its manufacturer, is not guaranteed or endorsed by the publisher.

## Footnotes

1 The SVAR is appealing for many reasons, including the fact that all the variables included in the SVAR system are treated as endogenous and interdependent. Furthermore, the model can capture the static and dynamic interdependencies present in the data series using a minimum set of restrictions. In addition, the SVAR model explicitly accounts for variations in the transmission of shocks among the variables.
